# Prostaglandin E_2_ Promotes Features of Replicative Senescence in Chronically Activated Human CD8+ T Cells

**DOI:** 10.1371/journal.pone.0099432

**Published:** 2014-06-11

**Authors:** Jennifer P. Chou, Christina M. Ramirez, Danielle M. Ryba, Megha P. Koduri, Rita B. Effros

**Affiliations:** 1 Department of Pathology & Laboratory Medicine, David Geffen School of Medicine, University of California Los Angeles, Los Angeles, California, United States of America; 2 UCLA AIDS Institute, David Geffen School of Medicine, University of California Los Angeles, Los Angeles, California, United States of America; 3 Department of Biostatistics, Fielding School of Public Health, University of California Los Angeles, Los Angeles, California, United States of America; New York University, United States of America

## Abstract

Prostaglandin E_2_ (PGE_2_), a pleiotropic immunomodulatory molecule, and its free radical catalyzed isoform, iso-PGE_2_, are frequently elevated in the context of cancer and chronic infection. Previous studies have documented the effects of PGE_2_ on the various CD4+ T cell functions, but little is known about its impact on cytotoxic CD8+ T lymphocytes, the immune cells responsible for eliminating virally infected and tumor cells. Here we provide the first demonstration of the dramatic effects of PGE_2_ on the progression of human CD8+ T cells toward replicative senescence, a terminal dysfunctional state associated multiple pathologies during aging and chronic HIV-1 infection. Our data show that exposure of chronically activated CD8+ T cells to physiological levels of PGE_2_ and iso-PGE_2_ promotes accelerated acquisition of markers of senescence, including loss of CD28 expression, increased expression of *p16* cell cycle inhibitor, reduced telomerase activity, telomere shortening and diminished production of key cytotoxic and survival cytokines. Moreover, the CD8+ T cells also produced higher levels of reactive oxygen species, suggesting that the resultant oxidative stress may have further enhanced telomere loss. Interestingly, we observed that even chronic activation *per se* resulted in increased CD8+ T cell production of PGE_2_, mediated by higher COX-2 activity, thus inducing a negative feedback loop that further inhibits effector function. Collectively, our data suggest that the elevated levels of PGE_2_ and iso-PGE_2_, seen in various cancers and HIV-1 infection, may accelerate progression of CD8+ T cells towards replicative senescence *in vivo*. Inhibition of COX-2 activity may, therefore, provide a strategy to counteract this effect.

## Introduction

Lipid mediators have long been recognized as key regulators of inflammation and homeostasis. Prostaglandins constitute one of the most important families of these mediators. In particular, prostaglandin E_2_ (PGE_2_), a common arachidonic acid-derived eicosanoid produced by cyclooxygenases (COX1 and COX2), is involved in a wide variety of physiological events. It is markedly increased during inflammatory processes, and it helps promote vasodilation; moreover, its chronic biological effects have been linked to the pathogenesis of certain malignancies and HIV disease. Within the immune system, PGE_2_ modulates such critical processes as cytokine production, differentiation, proliferation, migration and antigen presentation [1], [2].

Several pathologies suggest a role for PGE_2_ in specifically modulating the function of T cells. For example, CD8+ T cells from HIV-infected persons have increased intracellular cyclic AMP (cAMP), a downstream target of the PGE_2_ signaling cascade. Furthermore, elevated serum levels of prostaglandins correlate with worse clinical prognoses in HIV/AIDS [3], [4]. In addition, T cells from patients with PGE_2_-secreting cancers show decreased proliferation in response to anti-CD3 antibody stimulation [5]. Interestingly, aging in both mice and humans is associated with increased PGE_2_ secretion by activated macrophages, which could potentially impact responses of T cells in their proximity [6]. A great deal of the PGE_2_/immunology research has been focused on the development and differentiation of the CD4+ T cell subset, particularly in regard to its role in facilitating expansion of Th1 and Th17 cells [7], [8]. However, little is known about the effect of PGE_2_ on CD8+ T cells, for example, with respect to their progression towards replicative (cellular) senescence, a state of functional dysregulation and irreversible cell cycle arrest considered to be a contributor to failed immune responses during chronic infection and aging.

Oxidative stress, previously documented to increase the levels of PGE_2_ and its free-catalyzed isoform, iso-PGE_2_ [9], is also known to accelerate the process of replicative senescence. In this study, we addressed the question of whether PGE_2_ and iso-PGE_2_ themselves might have effects on replicative senescence that are distinct from those caused by oxidative stress. To address this question, we used a well-established *in vitro* model of T cell replicative senescence to measure changes in CD8+ T cell proliferation, telomerase activity, production of key cytokines, and expression of costimulatory molecules during chronic activation in the presence of these immunomodulators. Our data show that exposure to exogenous PGE_2_ and iso-PGE_2_ accelerates the senescence trajectory and associated effector functions of CD8+ T cells. Importantly, persistent, chronic stimulation of T cells *per se* increases COX-2 activity in CD8+ T cells, leading to endogenous production of PGE_2_. Our data suggest a mechanism by which cancer cells, aging and chronic infections may each contribute to T cell dysfunction and senescence.

## Materials and Methods

### Ethics Statement

All study participants for this study were recruited from the Los Angeles metropolitan area. This study was approved by the University of California, Los Angeles Medical Institutional Review Board and each participant provided written, informed consent per the approved protocol.

### Cell Cultures

Human peripheral blood samples from self-reported healthy donors were acquired by venipuncture after informed consent, and in accordance with the UCLA IRB. After centrifugation, the layer of peripheral blood mononuclear cells (PBMC) was carefully removed and washed twice in complete RPMI (5% fetal bovine serum, 10 mM Hepes, 2 mM glutamine, 50 IU/mL penicillin/streptomycin). The EasySep CD8+ enrichment kit (Miltenyi Biotec) was used to isolate CD8+ T cells by negative selection, and purity of the cells was verified by flow cytometry (routinely >90% CD8+). Cultures of purified T cell were established as described previously [10]. Briefly, CD8+ T cells were exposed to diluent (DMSO) or to 100 nM–1 µM PGE_2_, iso-PGE_2,_ the EP2 antagonist AH6809, EP4 antagonist CAY10598, or a COX-2 inhibitor CAY10404 (all from Cayman Chemical) for 30 minutes and then activated with anti-CD2/CD3/CD28 microbeads, used as surrogate antigen (Miltenyi Biotec) with 10 µl microbead cocktail added for every 1×10^6^ cells. Stimulation and the modulator pre-treatment were repeated every 14–17 days. In some experiments, 500 nM butaprost (EP2 agonist), 500 nM misoprostol (EP4, EP3> EP1> EP2 agonist; each from Cayman Chemical), 1 µM Forskolin or H89 dihydrochloride (both Tocris Bioscience) were added. Cultures were supplemented with recombinant IL-2 (20 U/mL). Every 3–4 days, viable cell concentration was determined by trypan blue exclusion, and when the concentration reached ≥8×10^5^/ml, cells were subcultivated to a density of 5×10^5^ cells/ml. Population doublings (PD) were determined according to the formula: PD = log_2_ (final cell concentration/initial cell concentration).

### Quantitative PCR

Gene expression was evaluated by quantitative polymerase chain reaction (qPCR) analysis. In brief, after extraction by RNeasy Mini kit (Qiagen), 500 ng of RNA from T cells was reverse-transcribed with the iScript cDNA synthesis kit (Bio-Rad). The qPCR assays were performed using the Bioline SensiFAST SYBR Kit and CFX 96 (Bio-Rad). The housekeeping gene, *36B4*, was used as an internal control. The sequences of the primers were designed using Primer 3 software, listed below. Samples were run in triplicate in a 96-well plate using the settings of 95°C for 2 minutes, 95°C for 5 s and 60°C for 15 min (single fluorescence measurement) with the 2^nd^ and 3^rd^ step repeated for 39 cycles. Primer sequences are listed on Table 1.

**Table 1:** List of primers.

36B4	F-5′-CAATCTGCAGACAGACACTGG-3′
	R-5′-TCTACAACCCTGAAGTGCTTGAT-3′
hTERT	F-5′-AAGTTCCTGCACTGGCTGATG-3′
	R-5′-GCTTTGCAACTTGCTCCAGAC-3′
CD28	F-5′-AGGCTCCTGCACAGTGACTA-3′
	R-5′-GAGCGATAGGCTGCGAAG-3′
IL-2	F-5′-TCACCAGGATGCTCACATTTAAGTTTT-3′
	R-5′-TTCCTCCAGAGGTTTGAGTTCTTCTTC-3′
CTLA-4	F-5′TGAGTTGACCTTCCTAGATGA-3′
	R-5′CTGGGTTCCGTTGCCTATGC-3′
IFN-G	F-5′TCTGAGACAATGAACGCTAC-3′
	R-5′GAGTAGGCTCACCAGGTG-3′
COX-2	F-5′TGCTTGTCTGGAACAACTGC-3′
	R-5′TGAGCATCTACGGTTTGCTG-3′
p16	F-5′ATATGCCTTCCCCCACTACC-3′
	R-5′CCCCTGAGCTTCCCTAGTTC-3′

### Flow Cytometry

Surface expression of CD28, CD8, and CD3 was examined by immunostaining and flow cytometry. Cells were incubated with fluorescently labeled anti-CD3, -CD8, -CD28, -CD25, fluorophore conjugated antibodies (BD Biosciences) at 4°C for 20 min, washed, and fixed in PBS containing 1% paraformaldehyde. Parallel samples were incubated with Ig isotype control antibody or secondary Abs (BD Biosciences). For intracellular staining, cells were stimulated for 5 hr with anti-CD2/CD3/CD28 microbeads with or without the immunomodulators, treated with Golgistop for 6 hours, permeabilized and stained with PE-anti-IFN-γ and FITC-anti-TNF-α antibodies using the Cytofix/Cytoperm Plus kit (BD Biosciences). All samples were analyzed on a FACSCalibur flow cytometer (Beckton Dickson). Fluorescence data from at least 25,000 cells were acquired. Analysis of data was performed using Cell Quest Pro (BD Biosciences).

### Telomerase Activity Measurements

Telomerase activity was determined using a modified version of the Telomerase Repeat Amplification Protocol (TRAP) as previously described [11]. Briefly, for each sample 1×10^6^ CD8+ cells were pelleted and washed twice with PBS. Cell pellets were lysed in 100 µL of M-PER Mammalian Protein Extraction Reagent (Pierce Biotechnology) and allowed to incubate on ice for 1 hour. To control for inter-sample cell number variance, samples were normalized according to nucleic acid concentration, which was determined using spectrophometric readings for dsDNA. The endogenous telomerase present in the cell extract adds telomeric repeats to the telomerase substrate (TS), a nontelomeric oligonucleotide. The extension products are then amplified several-fold by PCR carried out by Taq polymerase using a Cy-5-labeled forward primer (TS: 5′−/5Cy5/AATCCGTCGACGCAGAGTT) as a substrate for telomerase-mediated addition of TTAGGG repeats, and an anchored reverse primer (ACX: 5′-GCGCCGCTTACCCTTACCCTTACCCTAACC-3′). Each sample was mixed with 20 ul of Bromothenol Blue loading dye and 35 µl of sample+dye and was loaded and run at least twice using 10% non-denaturing PAGE in 1X TBE buffer. Gels were run first at 100V for 20 min, followed by approximately 250 V for 2 h. Gels were scanned on a STORM 865 (GE Healthcare,) and quantified using the software ImageQuant 5.2, which integrates signal intensity over the telomere length distribution on the gel as a function of molecular weight (GE Healthcare).

### PGE_2_ Measurements

Culture supernatants were harvested 72 h post-stimulation and analyzed for PGE_2_ using the PGE_2_ EIA ELISA kit (Enzo Life Sciences). All measurements were performed in triplicate wells and in accordance to manufacturer’s recommendations. The sensitivity for this kit is 13.4–2,500 pg/ml.

### Measurement of Telomere Length

Genomic DNA was extracted from CD8+ T cells using the DNeasy Tissue Kit according to manufacturer’s instructions (Qiagen). Real-Time PCR was performed on a total of 5 ng of DNA per sample using IQ Sybr Green Supermix according to the manufacturer’s instructions (Bio-Rad) and an established quantitative telomere PCR protocol [12]. The primers used for: Tel 1b: 3′-CGGTTTGTTTGGGTTTGGGTTTGGGTTTGGGTTTGGGTT-5′ and Tel 2b: 3′-GGCTTGCCTTACCCTTACCCTTACCCTTACCCTTACCCT-5′. HGB 1: 3′-GCTTCTGACACAACTGTGTTCACTAGC-5′ and HGB 2: 3′-CACCAACTTCATCCACGTTCACC-5′. Genomic DNA extracted from SAOS cells (Human osteocarcinoma cell line; American Type Culture Collection) with known telomere length was included in each PCR reaction to control for inter-assay variation and for comparison among donors. A no-template control was included in all PCR reactions, and data from all samples were expressed as a percentage of the telomere length of the tumor cell line, SAOS (∼23 kb), as described previously [13].

### Intracellular cAMP Determination

CD8+ T cells were cultured as described, and at 72 h or 14 d post stimulation with anti-CD2/CD3/CD28 microbeads, the cells were pelleted and washed twice with 1xPBS. Cells were then lysed during incubation for 20 min with HCl (0.1 M). Cell debris was removed by centrifugation at 600×g for 10 min, and the level of cAMP in the supernatants was determined using a direct cAMP enzyme immunoassay kit (Enzo Life Sciences), following the manufacturer’s protocol. Assays were performed in triplicate.

### COX-2 Enzyme Activity Assay

Changes to the enzymatic activity of COX-2 were measured using a COX enzyme activity assay (Cayman Chemical), according to the manufacturer’s protocol. This assay measures COX-2 activity by oxidation of the peroxidase cosubstrate TMPD (N,N,N1,N1-tetra-methyl-p-phenylenediamine) in 96-well plates and has been shown to accurately reflect the rate of conversion of arachidonic acid to PGH2. In brief, CD8+ T cells were collected 72 h or 14–17 d post Ab-bead activation. Cells were then rinsed twice in 1xPBS, lysed in ice-cold 0.1 M Tris (pH 7.8, 1 mM EDTA), and stored at −80C until the assay was performed. The assay mixture containing assay buffer, heme, sample (or protein standard prepared by boiling lysates), and the COX-1 inhibitor SC-560 to eliminate COX-1 activity (or as a control reaction the COX-2 inhibitor DuP-697 to eliminate COX-2 activity) was incubated for 5 min at 25°C, and TMPD was added. The reactions were initiated by adding arachidonic acid to all wells, and plates gently were shaken and incubated for 5 min at 25°C and the absorbance was then read at 590 nM. COX-2 activity was then calculated using the following formula whereby 1 unit is defined as the amount of enzyme to oxidize 1 nmol of TMPD per min at 25°C: COX-2 activity = [(Δ590/5 min/0.00826 µM-1) × (0.21 ml/0.01 ml)]/2 (it takes two molecules of TMPD to reduce PGG2 to PGH2) = nanomoles per minute per milliliter (units per milliliter).

### Intracellular ROS Measurement

Intracellular ROS were measured by flow cytometry using the fluorescent probe dichlorodihydrofluorescein diacetate (H2DCFDA, Molecular Probes), which is oxidized to highly fluorescent dichlorodihydrofluorescein (DCF) by hydroxides, hydrogen peroxides, and hydroxyl radicals. Briefly, T lymphocytes (1×10^6^/ml) were treated with PGE_2_ and iso-PGE_2_ for 24 h, and then incubated with DCFDA (2 µM) for 30 min at 37°C in the dark. At the end of incubation, cells were washed and resuspended in HBSS at 37°C. To determine the effects by mitochondrial ROS, MitoSOX Red–based flow cytometric detection of mitochondrial superoxide was used. Cells were incubated with MitoSOX Red superoxide indicator (Invitrogen) for 30 min and washed, and PGE_2_ and iso-PGE_2_ were added for 2 h. The cells were analyzed on a FACSCalibur (BD Biosciences). Analyses of data were performed using Cell Quest Pro (BD Biosciences).

### Statistical Analysis

Mean values and standard deviation as well as medians and IQRs were calculated for each time-point. Significance was established by the Kruskall Wallis test for between group comparisons, a non-parametric test similar to ANOVA using SAS V. 9.2 (SAS Institute, Cary, NC). For data where each donor had a control sample and a treatment sample, differences were used so that each donor could serve as his or her own control. Differences for these data were assessed using the nonparametric permutation test for paired data; p values of <0.05 were considered significant.

## Results

### CD8+ T Cells Upregulate Prostaglandin Receptors upon Activation and are Sensitive to PGE_2_ and iso-PGE_2_

**Figure 1:**
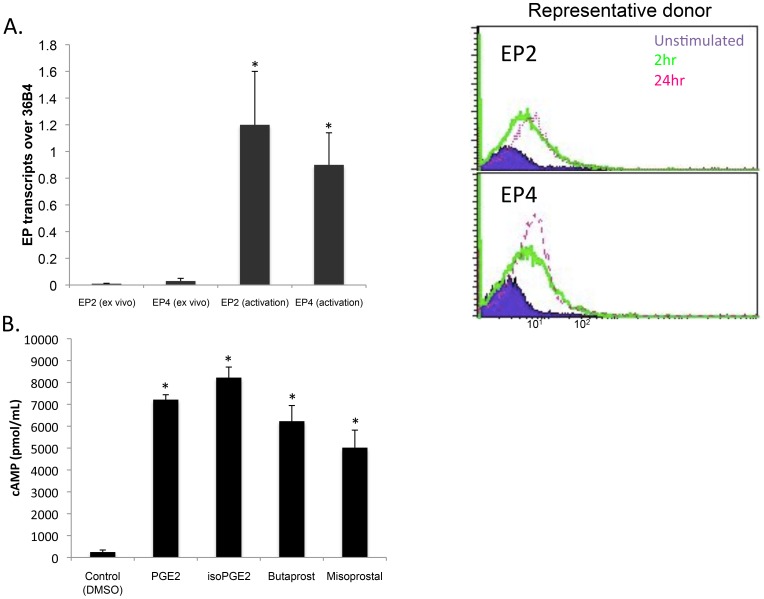
EP receptor expression and cAMP upregulation in CD8+ T cells. CD8+ T cells were freshly isolated from PBMC from whole blood derived from healthy donors or HIV+ persons. (A) (Left) *EP2* and *EP4* transcripts were evaluated by quantitative PCR in *ex vivo* samples and in T cells activated with anti-CD2/CD3/CD28 microbeads for 24 hours. *36B4* was used as the housekeeping gene and data represents 3 healthy donors performed on a single plate (*p = 0.05). (Right) EP2 and EP4 surface expression was also evaluated in healthy at 2 hours and 24 hours post activation, or with no Ab-coated bead activation. Flow cytometric histogram shows one representative donor from a healthy person stained with PE–anti-human EP2 or PE–anti-EP receptor antibodies (Cayman Chemical). (B) Intracellular cAMP was evaluated using a direct cAMP ELISA kit (Enzo Biosceinces) in T cells treated with PGE_2_, isoPGE_2_, and known EP agonists, misoprostol (EP2, EP3, and EP4) and butaprost (EP2) for 72 h (n = 3; p<0.005 by Kruskal Wallis for the comparison of all treatment groups to control).

In order to initiate studies on the specific CD8+ T cell effects of PGE_2_, it was first necessary to confirm that this subset expresses the same PGE_2_-specific receptors previously reported for the total CD3+ T cell population, namely EP2 and EP4 [7]. Figure 1A shows that CD8+ T cells from peripheral blood of healthy donors upregulated *EP2* and *EP4* mRNA and protein upon activation with anti-CD2/CD3/CD28 microbeads, with no evidence of expression when tested immediately *ex vivo*. These observations suggest that T cell receptor (TCR) and CD28 engagement is required for the upregulation of the EP receptors. Consistent with previous reports, no transcripts of *EP1* and *EP3*, the other known EP receptors, were observed after activation (data not shown). Therefore, these results indicate that EP2 and EP4 constitute the major PGE_2_ receptors on CD8+ T cells. If similarly enhanced receptor expression occurs *in vivo*, CD8+ T cells may show increased sensitivity to the effects of PGE_2_ in immune-suppressed HIV+ persons.

PGE_2_ has been reported to modulate function of murine T cells and the human Jurkat T cell tumor line via cAMP-PKA signaling [14], [15]. The data in Figure 1B extend these findings to normal human CD8+ T cells, showing that inclusion of 500 nM PGE_2_ and iso-PGE_2_ in cell culture results in a significant increase in intracellular cAMP, which peaked shortly after exposure and remained elevated even after 72 hours. T cells treated with butaprost (500 nM) and misoprostal (500 nM), which are agonists for EP2 and EP2/EP4, respectively, also showed similar levels of intracellular cAMP (Fig. 1B). These results indicate that human CD8+ T cells are able to recognize and be affected by PGE_2_ and its free-catalyzed isoform, iso-PGE_2_.

### PGE_2_ and iso-PGE_2_ Decrease Proliferative Potential and Increase the Transcription of the *p16* Cell Cycle Arrest Gene

The ability to rapidly expand *in vivo* upon TCR and CD28 engagement is central to T cell function and is crucial for an effective immune response. To assess the effects of PGE_2_ and iso-PGE_2_ on CD8+ T cell proliferative potential, we measured the total number of population doublings (PD) of CD8+ T cell cultures that are driven to the end stage of replicative senescence following multiple rounds of chronic activation, as described previously [16], [17]. The end stage of replicative senescence is experimentally defined as the inability of CD8+ T cells to enter cell cycle in response to two rounds of stimulation, and coincides with several functional changes, such as loss of telomerase activity and surface expression of CD28, an important costimulatory molecule [18].

**Figure 2:**
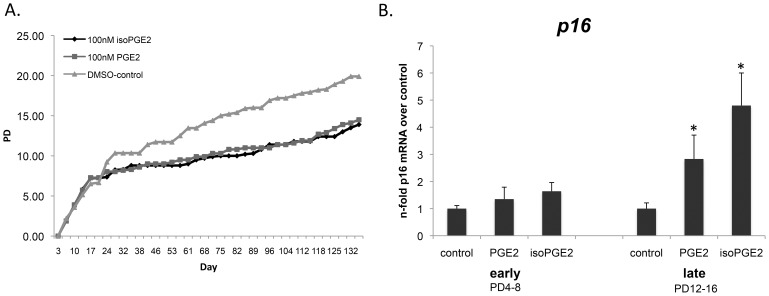
Proliferative potential decreases and *p16* transcripts increase in the presence of PGE_2_ and iso-PGE_2_. T cells were activated with anti-CD2/CD3/CD28 microbeads with or without PGE_2_ or iso-PGE_2_ and population doublings (PD) was calculated by the formula PD = log_2_ (final cell concentration/initial cell concentration) (A) Long term culture of one representative donor. (B) *p16* transcripts were quantified by qPCR during early (PD4–8) and late (PD12–16) time points in the presence of PGE_2_, iso-PGE_2_ or diluent (n = 5; *p = 0.031 compared to control by the paired permutation test). *36B4* was used as the housekeeping gene.

Using this cell culture protocol, we found that a 30-minute pre-treatment with physiological concentrations of the immunomodulators (0.05–1 µM PGE_2_ or iso-PGE_2_) prior to each round of activation decreased the total PD in a dose-dependent manner. PGE_2_ and iso-PGE_2_ treated T cells maximally reached a total PD 14–16 versus 20–24 observed in diluent (DMSO)-treated cultures (Fig. 2A). All donors followed a similar pattern of growth when treated with PGE_2_ or iso-PGE_2_, with observed PDs 35–60% less than control cultures. Furthermore, we tested the effects of PGE_2_ and iso-PGE_2_ on proliferation and metabolic activity of T cells using an MTT assay (data not shown), and found that a 4hr incubation with the immunomodulators decreased absorbance at 570 nm by an average of 31% among three donors.

Upregulation of the cyclin dependent kinase inhibitor, p16, is a major mediator of senescence in many cell types [17], [19]. Our data demonstrate that CD2/CD3/CD28 activation in the presence of PGE_2_ and iso-PGE_2_ significantly increases *p16* transcripts during the later phases of the culture, i.e., 14–18 PD (p = 0.0315 for both PGE_2_ and iso-PGE_2_) (Fig. 2B). This upregulation of the cell-cycle arrest marker leading to premature induction of senescence-like characteristics, if occurring *in vivo,* would presumably reduce the ability of activated CD8+ T cells to expand and mount a vigorous offense against pathogens and tumor cells.

### PGE_2_ and iso-PGE_2_ Induce Premature Replicative Senescence: Telomeres and Telomerase Activity

**Figure 3:**
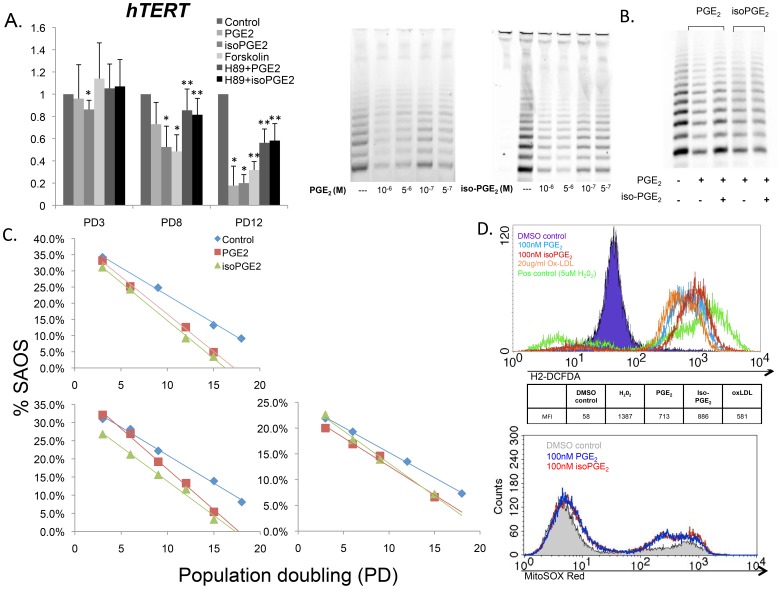
PGE_2_ and iso-PGE_2_ inhibit telomerase activity while increasing intracellular ROS. CD8+ T cells were negatively selected after 72 hours post activation with anti-CD2/CD3/CD28 microbeads with or without PGE_2_ or iso-PGE_2_, and inhibitors of PKA pathway. (A) (Top Left) *hTERT* expression was quantified at equivalent PDs by qPCR (n = 5; *p = 0.0312; **p = 0.031 using one-sided T test because of decreased sample size for these conditions only; n = 4). (Top Middle) Representative gel showing effects of PGE_2_ (10^−6^ to 5^−7^M) and iso-PGE_2_ (10^−6^ to 5^−7^M) on telomerase activity of CD8+ T cells. Band intensity per lane correlates with relative telomerase activity of 1,250 CD8+ T cells in each treatment group. (B) Telomerase activity was measured as described in (A) with PGE_2_ or iso-PGE_2_ in the presence of 1 µM of a PKA inhibitor, H89 dihydrochloride. (C) CD8+ T cell cultures were established and chronically activated as previously described in the presence of the immune modulators. Telomere lengths were evaluated by Real-Time PCR and expressed as a percentage of telomere length of a human tumor cell line, SAOS (∼23Kb). Data represent telomere lengths over the lifetime from 3 representative donor cultures. (D) (Top) The relative amount of intracellular ROS was determined by the mean fluorescence intensity of DCFDA–stained, live CD8+ T cells after 24 h of culture with media alone or in the presence of PGE_2_, isoPGE_2_, ox-LDL, or H_2_O_2_ (pos control). (Bottom) Representative flow cytometry profile of MitoSOX red oxidation in PGE_2_- and iso-PGE_2_-treated T cells.

One of the signature features of T cell replicative senescence is the loss in activity of telomerase, a holoenzyme that extends the protective ends of chromosomes called telomeres [20]. The induction of telomerase is essential for T cell proliferation and memory T cell maintenance during infection. Loss of telomerase in CD8+ T cells is also predictive of more rapid pathogenesis and worse clinical outcomes in HIV disease, several cancers, age-related bone disorders, and a host of other pathologies [21], [22]. We therefore evaluated the effects of PGE_2_ and iso-PGE_2_ on telomerase activity and telomere length of CD8+ T cells over time. The data in Figure 3A document that after the first two rounds of activation, there was a dose-dependent decrease in both transcription of *hTERT*, the telomerase catalytic subunit, and actual telomerase activity in cultures exposed to PGE_2_ and iso-PGE_2_. To elucidate the underlying mechanism for this downregulation, we treated the T cells with forskolin, a potent stimulator of the cAMP-PKA pathway, and observed a similar pattern of hTERT message reduction (Fig. 3A) and telomerase activity (data not shown). In addition, H89, an inhibitor of PKA, modestly restored some telomerase activity when pre-incubated with the cells prior to PGE_2_ or iso-PGE_2_ addition (Fig. 3B).

As expected, the reduction of telomerase activity was associated with telomere shortening in the PGE_2_- and iso-PGE_2_-treated cells. The shorter telomere lengths in the treated vs. control cultures were evident both after the same number of days in culture as well as after identical numbers of PD, illustrated in one representative donor (Fig. 3C). Critically short telomeres may cause the T cells to abruptly enter permanent cell-cycle arrest, consistent with the observed increase of *p16* transcripts (Fig. 2B). Given that increased oxidative stress is a known inducer of cellular senescence, we tested the possibility that PGE_2_ and iso-PGE_2_ may be contributing to accelerated telomere shortening in the T cells via induction of reactive oxygen species (ROS). Indeed, increased mean fluorescence intensity of CD8+ T cells labeled with ROS-sensitive dye DCFDA was observed in the presence of both modulators, but not in the control cultures (Fig. 3D), reaching levels comparable to those caused by H_2_O_2_ treatment. The MitoSOX red dye was used to detect mitochondrial superoxide, another free radical species thought to contribute to DNA damage and senescence. Oxidation of MitoSOX red was observed to be modestly higher in PGE_2_ and iso-PGE_2_ CD8+ T cells (Fig. 3D), indicating that chronic exposure to these modulators can increase ROS production and contribute to the development of dysfunctional phenotypes in T cells.

### Key Features of T Cell Function are Modulated by PGE_2_ and iso-PGE_2_

**Figure 4:**
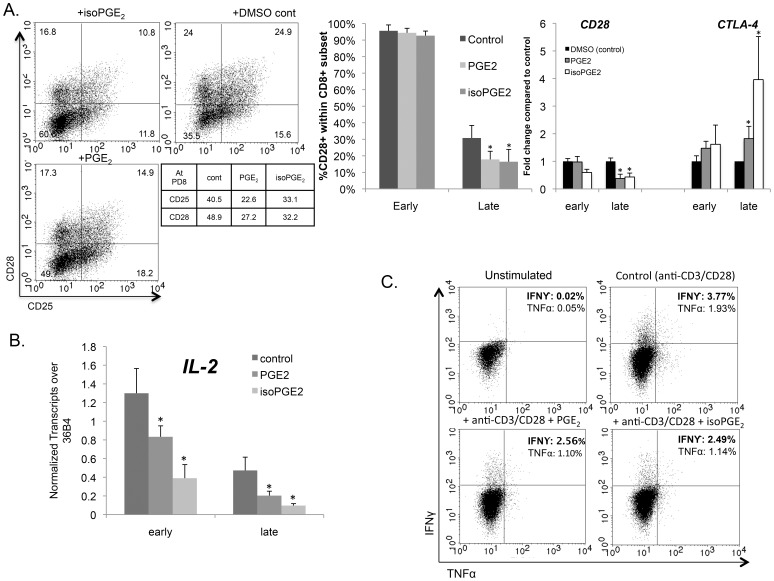
Key features of T cell function are modulated by PGE_2_ and isoPGE_2_. (A) (Top Left) CD8+ T cells treated with iso-PGE_2_ (top) and PGE_2_ (bottom) were immunostained with FITC–anti-CD25, PE–anti-CD28, and gated on PerCP–anti-CD8 and APC–anti-CD3+ T cells (all BD Biosciences). Samples were compared at the same PD. (Top Middle) The %CD28+cells from different treatment samples over the lifetime of the culture in five donors; *p = 0.031 (Top right) *CD28* and *CTLA-4* expression was determined by qPCR during both early (PD4–8) and late (PD12–16) culture stages in CD8+ T cells treated with the immunomodulators (n = 5; *p = 0.031). All samples were tested in triplicate and normalized to the housekeeping gene *36B4* (B) *IL-2* message was similarly quantified by qPCR as in (A) after treatment (n = 5; *p = 0.031) (C) CD8+ T cells were stimulated with Ab-coated microbeads for 72 h in the presence of PGE_2_, iso-PGE_2,_ or were treated with diluent (DMSO). Intracellular IFN-γ and TNF-α was analyzed flow cytometrically using FITC–anti-TNF-α, PE–anti–IFN-γ, PerCP–anti-CD8 and APC–anti-CD3. The frequencies of the IFN-γ– and TNF-α producing cells in T-cell fractions gated on CD3+CD8+ are shown as percentages.

The T cell co-stimulatory receptor, CD28, provides an important second signal that is necessary for a robust activation through the T cell receptor, promoting cell expansion while preventing the induction of anergy or cell death [23]. Since loss of CD28 gene and surface expression is a key feature of replicative senescence, the effects of PGE_2_ and its free-catalyzed isoform on CD28 were evaluated. We observed that CD28 surface expression and *CD28* transcripts were reduced in the presence of PGE_2_ and iso-PGE_2_, with pronounced effects after the third round of activation. In the representative experiment shown in Fig. 4A (left panel), compared to DMSO-controls, which were nearly 49% CD28+, the PGE_2_- and iso-PGE_2_-treated CD8+ T cells were only ∼32% and ∼28% CD28+, respectively. Representative time courses for three cultures show that loss of CD28 surface expression was more rapid in the treated cultures than in controls (Fig. 4A). These results may explain the reduced telomerase activity described above, since CD28 has been shown to play a direct regulatory role on gene expression of *TERT*, the catalytic subunit of telomerase, and telomerase activity [10].

Concomitant with this decline in CD28 expression was a detectable increase in gene expression of *CTLA-4*, a transiently expressed antigen that competes with CD28 for its binding partner, B7, on antigen-presenting cells (APCs). CTLA-4 delivers an inhibitory signal in activated T cells and thereby downregulates T cell function and expansion [23], [24]. Even after 72 hours post-activation in the presence of PGE_2_ or iso-PGE_2_, *CTLA-4* transcripts remained significantly higher than controls by 1.5–2 fold for PGE_2_ and 1.5–4 fold for iso-PGE_2_ (Fig. 4A - top right).

Senescence is also marked by the loss of IL-2, a cytokine that promotes T cell survival and differentiation into effector T cells [17]. In HIV/AIDS, IL-2 production is frequently used to assess T cell immunity since IL-2 producing CD8+ T cells are found in very low frequencies in viremic individuals progressing rapidly to AIDS, compared to long-term non-progressors [13]. Furthermore, HIV-1-specific IFN-γ/IL-2-secreting CD8+ T cells support CD4-independent proliferation of HIV-1-specific CD8+ T cells [25]. Figure 4 illustrates the significant downregulation of *IL-2* mRNA in the presence of PGE_2_ and iso-PGE_2_ during the early and late phases of the cultures (Fig. 4B) and an observed downregulation of the IL-2 receptor, CD25 (Fig. 4A). Studies have indicated that dysfunctional HIV-specific T cells with features of senescence lack responsiveness to exogenous IL-2 [26] and the downregulation of CD25 may contribute to their inability to actively expand *in vivo* [27], [28].

Another important cytokine that is critical for effector function, and whose loss is associated with senescence, is IFN-γ, a potent and multifunctional anti-viral effector molecule that is readily secreted by CD8+ T cells upon recognition of foreign peptide presented on HLA class I molecules. In metastatic cancers and chronic HIV infection, loss of IFN-γ-producing T cells correlates with more rapid disease progression and worse clinical outcomes [29], [30], [31]. Consistent with reports on the role of prostaglandins on lymphocytes, PGE_2_ and iso-PGE_2_ significantly downregulated IFN-γ, measured both in the form of intracellular protein expression (Fig. 4C) and transcript abundance (data not shown). Also in accord with previous reports [8], TNF-α cytokine production was decreased in the presence of the modulators (Fig. 4C). These data demonstrate significant impairments in CD8+ T cell function caused by PGE_2_ and iso-PGE_2_, ranging from the cells’ ability to express the costimulatory molecule CD28 to the secretion of important anti-viral cytokines. Taken together, these data suggest that PGE_2_ and iso-PGE_2_ promote the acquisition of multiple senescent features and may therefore play a key role in the accumulation of dysfunctional CD8+ T cells seen in chronic infections and cancer.

### COX-2 Activity and EP4 Expression Increases in T cells during Chronic Activation

The data presented thus far have focused on the effects of exogenous PGE_2_ and iso-PGE_2_ on repeatedly activated CD8+ T cells. However, we wondered whether the chronic activation in itself might result in increased production of PGE_2_, mediated by COX-2, which synthesizes PGE_2_ from arachidonic acid released from the plasma membrane. This possibility would be consistent with a report documenting observed elevated intracellular cAMP and the fact that COX-2 inhibitor therapy reduces features of immune dysfunction and exhaustion in CD8+ T cells in HIV-infected persons [3]. We therefore examined *COX-2* gene expression and activity in chronically activated T cells.

**Figure 5:**
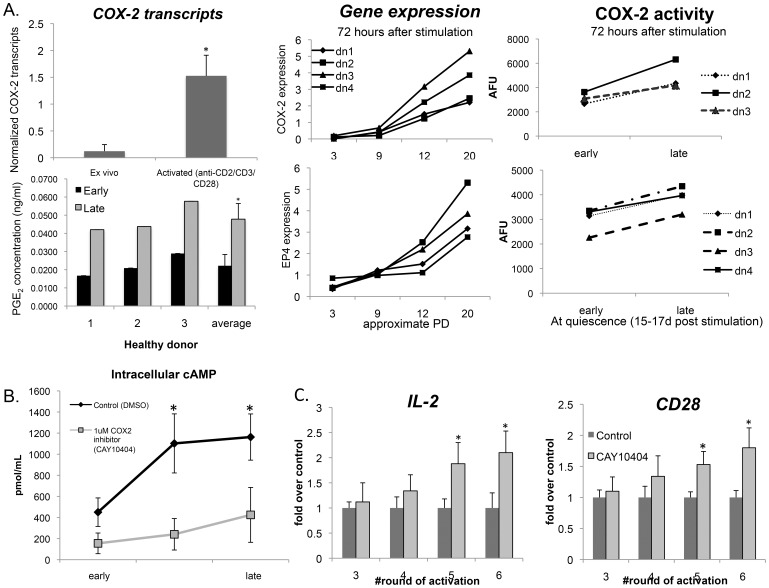
COX-2 activity and EP4 expression increases in T cells during chronic activation. (A) (Top left) *COX-2* transcripts were quantified by qPCR in *ex vivo* and activated (anti-CD2/CD3/CD28) CD8+ T cell samples from healthy donors. Each sample was tested in triplicate and normalized to the housekeeping gene *36B4* (n = 5; *p = 0.031). Supernatants from early and late cultures from 3 donors were collected 72 h post activation with Ab-coated microbeads and their average PGE_2_ concentration was calculated from triplicate wells, *p = 0.05. (Top Right) *COX-2* and *EP4* transcripts were quantified 72 h post each round of activation in four healthy donors. Each sample was tested in triplicate and normalized to the housekeeping gene, *36B4*. In addition, COX-2 activity was determined in CD8+ T cells at early and late time points 72 h post activation and at quiescence (15–17 d post stimulation) in three healthy donors using the COX Activity Assay (Cayman Chemical). (B) CD8+ T cells were pre-incubated with a highly specific COX-2 inhibitor CAY10404 (1 µM) (Cayman Chemical) or diluent (DMSO) for 30 min and then activated with Ab-coated microbeads as described. 1×10^6^ CD8+ T cells were collected 24h post activation during early and late PDs and intracellular cAMP was measured in triplicate using a direct cAMP ELISA kit (Enzo Life Sciences) (n = 4; *p = 0.05). (C) CD8+ T cells were cultured in the presence of 1 µM CAY10404 or DMSO for 30 min, and then activated with Ab-coated microbeads. 72 h after each round of activation, cells were collected and *IL-2* and *CD28* transcripts were quantified by qPCR. Each sample was tested in triplicate and normalized to the housekeeping gene *36B4* (n = 5; *p = 0.031).

Figure 5A shows that in cultures from healthy donors, there was a dramatic increase in *COX-2* transcripts 24 hours after CD2/CD3/CD28 engagement. Furthermore, repeated rounds of stimulation were associated with progressively increasing expression of *COX-2* and *EP4* transcripts, COX-2 activity and PGE_2_ in culture supernatants (Fig. 5A). Elevated intracellular cAMP (Fig. 5B – control cultures) was also detected in these “older” T cell cultures, suggesting that the modulation of the cAMP-PKA signaling pathway by TCR and CD28 engagement may significantly change during chronic activation, as seen in cancer, HIV infection and aging.

### COX2 Inhibition may Prevent the Development of Features of CD8+ T Cell Dysfunction

The data shown in Fig. 5A suggest that upregulation of COX-2 activity and subsequent increase in secreted PGE_2_ during chronic T cell activation may contribute to the development of features of replicative senescence associated with persistent infections, cancer and aging. To address one potential therapeutic approach, we investigated whether COX-2 inhibition in mid-to-late culture (i.e. PD12–20) would retard some of the features of replicative senescence described above. T cells were activated as previously described after a 30 min pretreatment with the highly specific COX-2 inhibitor, CAY10404. The specificity and activity of the COX-2 inhibition was first validated by measuring intracellular cAMP in CD8+ T cells after TCR and CD28 engagement (Fig. 5B). Pretreatment of CD8+ T cells with CAY10404 resulted in higher mean levels of *CD28* and *IL-2* transcripts (2-fold and 1.8-fold, respectively, over DMSO-control at the 6^th^ round of stimulation) (Fig. 5C). The data suggest that endogenous COX-2 activity and production of PGE_2_ may contribute to the development of immune senescence, and that reducing COX-2 activity may retard some of its features. Together, these results suggest potential therapeutic benefits of COX-2 inhibition in slowing the senescence trajectory of CD8+ T cells that arise during chronic activation.

## Discussion

The current study represents the first report documenting the effects of PGE_2_ and iso-PGE_2_ on the senescence trajectory of human CD8+ T cells, in particular with regard to their CD28 expression, IL-2 transcription and telomerase activity. It provides a potential mechanism by which cancer cells, aged APCs, and HIV infection promote immune dysfunction and inefficient surveillance during chronic activation. To our knowledge, this is also the first documentation of the cAMP-PKA pathway in modulating telomerase and CD28 expression. One recent study found that this signaling cascade is a regulator of IL-2 expression [32], but it was unclear how the critical players of the cAMP-PKA pathway, which cross talks with such pathways as NFAT and MAPK/ERK, affect expression of hTERT and CD28. Interestingly, it has been reported that PKA increases phosphorylation of the Wilms tumor suppressor (WT1) protein, a potent transcriptional repressor that inhibits hTERT expression by direct binding to the hTERT promoter. This observation suggests that the effects of PGE_2_ can also be a result of its ablation of IL-2 signaling via blockade of JAK3 activation [32], thereby suppressing the cell’s ability to proliferate, which would then lead to the loss of telomerase.

In the course of these experiments, we also observed that pretreatment of CD8+ T cells with PGE_2_ and iso-PGE_2_ leads to a marked reduction of surface expression of IL-7R (CD127) during chronic activation (data not shown). The presence of CD127 on CD8+ T cells during the antiviral immune responses is thought to be a biomarker of effector T cells that successfully mature into highly proliferative protective memory T cells [33], [34]. Thus, loss of CD127 expression would be detrimental to long-term memory T cell maintenance and immune surveillance. In addition, many of the PGE_2_-associated inhibitory effects on CD8+ T cells including reductions in *hTERT* transcription, telomerase activity, and proliferative potential, were even more pronounced by its free-catalyzed isoform, iso-PGE_2_. This highlights a potential avenue by which free radicals, which can directly induce the peroxidation of arachidonic acid in the lipid membrane to produce this isoprostane, may weaken effector T cell functions and proliferation.

An unexpected finding of our study was that chronic activation itself amplifies COX-2 activity and production of PGE_2_. If this scenario occurs *in vivo*, the secreted PGE_2_ could directly interact with cell populations–including those of the immune system–within the local microenvironment. Although other cell types, such as myeloid and stromal cells, secrete the major portion of PGE_2_ *in vivo*, the production of this small-molecule derivative by T cells can affect other cells in a paracrine manner, possibly inducing maturation of dendritic cells and promoting active inflammation through its role as a vasodilator [8]. Indeed, the upregulation of COX-2 activity by T cells may enhance certain aspects of innate immune responses while dampening others; the autologously secreted PGE_2_ may also function in a negative feedback fashion to inhibit normal CD8+ T cell effector functions [8], [35]. Interestingly, increased PGE_2_ was recently detected in cervical tissue samples from women who were HIV-1 positive [36], implicating a potential causative relationship between chronic activation and PGE_2_ production by a variety of cell types. Clearly, COX-2 and PGE_2_ play a complex, sometimes paradoxical role, in immunity. Future *in vivo* studies will clearly be required in order to define the role of PGE_2_ secretion on and by T cells, and its effects on APCs and inflammation during chronic activation.

Finally, our study began to address possible therapeutic strategies to diminish the deleterious *in vivo* effects of PGE_2._ Since COX-2 activity was found to increase with each round of T cell activation, it seemed that COX-2 inhibition might be a promising approach to enhance T cell function, while simultaneously inhibiting secretion of PGE_2_ by certain tumor cells. Although our data (Fig. 5C) support this notion, clinical observations regarding the negative cardiac effects of the widely prescribed COX-2 inhibitor, celecoxib, suggest that other methods might be preferable for enhancing immunity. For example, blockade via knockdown or antagonists of alternative targets, including the major receptors, EP2 and EP4, or Microsomal prostaglandin E2 synthase-1 (mPGES-1), which catalyzes the formation of PGE_2_ from PGH_2_ downstream of COX-2, may be alternative therapeutic strategies to prevent accelerated acquisition of senescent features in T cells. Future *in vitro* and *in vivo* studies should clarify the utility and safety of the EP receptor or mPGES-1 blockade as therapeutic targets.
