# Correction: Prostaglandin E_2_ Promotes Features of Replicative Senescence in Chronically Activated Human CD8+ T Cells

**DOI:** 10.1371/journal.pone.0107600

**Published:** 2014-09-08

**Authors:** The PLOS ONE Staff

There are errors in Figure 3(B). The authors have provided a corrected version here.

**Figure 3:**
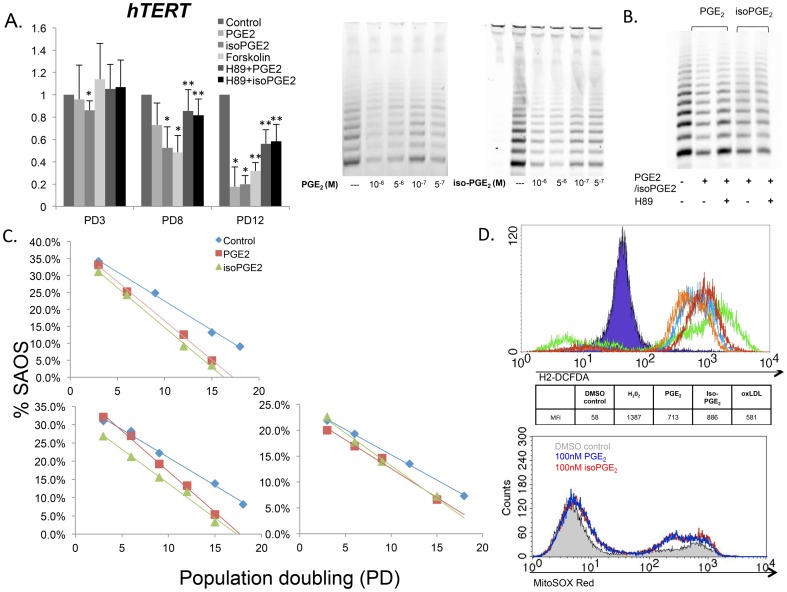
PGE_2_ and iso-PGE_2_ inhibit telomerase activity while increasing intracellular ROS. CD8+ T cells were negatively selected after 72 hours post activation with anti-CD2/CD3/CD28 microbeads with or without PGE_2_ or iso-PGE_2_, and inhibitors of PKA pathway. (A) (Top Left) *hTERT*expression was quantified at equivalent PDs by qPCR (n  =  5; *p  =  0.0312; **p  =  0.031 using one-sided T test because of decreased sample size for these conditions only; n  =  4). (Top Middle) Representative gel showing effects of PGE_2_ (10^−6^ to 5^−7^M) and iso-PGE_2_ (10^−6^ to 5^−7^M) on telomerase activity of CD8+ T cells. Band intensity per lane correlates with relative telomerase activity of 1,250 CD8+ T cells in each treatment group. (B) Telomerase activity was measured as described in (A) with PGE_2_ or iso-PGE_2_ in the presence of 1 µM of a PKA inhibitor, H89 dihydrochloride. (C) CD8+ T cell cultures were established and chronically activated as previously described in the presence of the immune modulators. Telomere lengths were evaluated by Real-Time PCR and expressed as a percentage of telomere length of a human tumor cell line, SAOS (∼23Kb). Data represent telomere lengths over the lifetime from 3 representative donor cultures. (D) (Top) The relative amount of intracellular ROS was determined by the mean fluorescence intensity of DCFDA–stained, live CD8+ T cells after 24 h of culture with media alone or in the presence of PGE_2_, isoPGE_2_, ox-LDL, or H_2_O_2_ (pos control). (Bottom) Representative flow cytometry profile of MitoSOX red oxidation in PGE_2_- and iso-PGE_2_-treated T cells.
